# Bridging the Bond: High-Sensitivity External Printed Strain Sensors for Condition Monitoring of Adhesive Joints

**DOI:** 10.3390/s26123738

**Published:** 2026-06-11

**Authors:** Valentin Wilhelm Mauersberger, Björn Senf, Sandra Menzel

**Affiliations:** Fraunhofer Institute for Machine Tools and Forming Technology IWU, Nöthnitzer Straße 44, 01187 Dresden, Germany; bjoern.senf@iwu.fraunhofer.de (B.S.); sandra.menzel@iwu.fraunhofer.de (S.M.)

**Keywords:** adhesive bonding, adhesive joints, structural health monitoring, printed electronics, printed sensors, jet dispensing, strain sensors, strain gauge, carbon fiber reinforced plastic, CFRP

## Abstract

**Highlights:**

**What are the main findings?**

- External printed strain sensors spanning the adhesive gap achieve an average relative resistance change of 65.3% near the failure load, an order of magnitude higher than internal bond-line sensors with 6.6%.
- At the same time, the coefficient of variation increases from 7.6% for internal to 32.6% for external sensors and is largely influenced by adhesive-gap geometry.

**What are the implications of the main findings?**

- The high sensor signals, which exceed environmental drift levels of up to 19% reported for printed electronics, enable in-service condition monitoring of adhesive joints.
- A reproducible adhesive-gap geometry achieved by automated adhesive application, together with quality control of the printing process, is essential for industrial integration.

**Abstract:**

Adhesive joints typically require high safety factors, as their mechanical performance is highly sensitive to environmental and manufacturing variations. Health monitoring can reduce these safety factors by continuously assessing the condition of the joint. While intrinsic and extrinsic sensing approaches exist, they are often based on periodic inspection or manual sensor integration, which limits their suitability for continuous in-service monitoring. This study investigates a novel sensor placement using additively manufactured strain sensors deposited by jet dispensing across the adhesive gap. Tensile lap-shear specimens were fabricated using CFRP (carbon-fiber-reinforced plastic) laminate, an epoxy adhesive, and silver-ink strain sensors placed internally within the joint and externally across the adhesive gap. Mechanical testing revealed that externally printed sensors produced an average resistance change of 65.3% near the failure stress of the adhesive joint, an order of magnitude higher than sensors embedded within the adhesive layer with 6.6% average resistance change. However, the average coefficient of variation increased as well, from 7.6% for internal to 32.6% for external. This sensor response exceeds reported environmentally induced variations in printed sensors and thus represents a promising candidate for condition monitoring. Further work is required to demonstrate actual damage detection capabilities and assess long-term stability under environmental and cyclic loading conditions.

## 1. Introduction

Adhesive joints are currently designed with significant safety margins because environmental stressors such as humidity, temperature fluctuations and aging or manufacturing variations including kissing bonds, voids, porosity, improper curing, and substrate-related failures can degrade their strength and thereby reduce the service life of the structure. By monitoring the structural health of adhesive joints and facilitating advanced modeling approaches, over-dimensioning can be avoided, thus enabling lightweight designs [1]. Traditionally, structural health monitoring of adhesive joints relies on established extrinsic non-destructive testing techniques such as ultrasonic inspection, acoustic emission, thermography, or vibration-based methods [2]. However, these approaches are often limited by their sensitivity, high equipment cost, or the need for periodic manual inspections rather than continuous in-service monitoring, which increases maintenance effort. Newer intrinsic methods involve sensors embedded directly within the bond line. Fiber optic sensors, piezoelectric and nanocomposite-based conductive networks allow for real-time detection of damage initiation and progression at the interface [2]. In this context, sensor placement plays a crucial role in the effectiveness of structural health monitoring [3]. A predominantly research-oriented approach for achieving extrinsic continuous in-service monitoring is back-face strain measurement. In this method, the strain gauges are applied to the outer surfaces of the adherends in the load-transfer region, allowing them to capture the local mechanical strain field of the adhesive joint [4,5,6]. When a crack initiates within the adhesive layer, the crack growth moves the stress distribution, which in turn leads to changes in the local strain field. These changes can be detected in the electrical resistance of the strain gauges [7].

The installation of strain gauges and intrinsic sensors is a predominantly manual process, particularly for low production volumes, and it requires careful surface preparation, precise alignment and adhesive application [8]. Additive manufacturing technologies, such as aerosol jetting, inkjet printing, direct-ink-writing, micro-pipette or jet dispensing, offer an alternative by depositing the sensing structures directly onto the component surface in an additive process. These approaches eliminate the manual installation required by conventional gauges and represent a key advantage by enabling customized sensor geometries and the fabrication of conformal sensors on curved surfaces [9]. They utilize materials consisting of a structural matrix filled with conductive additives. Recent developments have focused on nanocomposite systems, where carbon nanotubes and graphene are embedded in polymers, elastomers or resins. While offering high flexibility, tunable sensing behavior and a gauge factor (GF) ranging from single-digit values comparable to conventional strain gauges up to several hundred or even thousands, these materials exhibit complex sensing behavior with regions of different gauge factor due to different mechanisms of electric charge transport [10,11]. More traditional and still widely used approaches rely on inks with conductive additives such as silver, gold or carbon particles, with silver-based inks being the most common [12]. After depositing these inks, a sintering process is often needed to remove matrix material and improve the electrical properties of the sensor [13]. The sintering temperature and thus the electrical properties of the sensor are limited by the base structure onto which the sensor is printed. Compared to nanocomposite systems, the filler content of the inks is significantly higher. As a result, a denser and therefore more stable conductive network forms after deposition and curing. Consequently, the sensing mechanism is dominated by geometrical changes, leading to a mostly linear sensor response [14,15]. As shown in the investigations by Kravchuk and Reichenberger [16], a gauge factor (GF) of 2.52 can be achieved at a sintering temperature of 180 °C, while a GF of 4.29 can be achieved at 300 °C. Compared to a conventional strain gauge with a GF of 2–2.1, stronger sensor responses are possible with printed sensors, depending on the sintering temperature [16]. However, printed sensors show significantly higher scattering when exposed to environmental influences; the resistance of the sensors changes by up to 19% when stored at elevated temperatures and humidity [16]. In addition, sensor-to-sensor resistance is influenced by variations in the geometry of the printed conductive tracks. In jet dispensing processes, such geometric variations depend on numerous machine- and material-specific process parameters and therefore require paste-specific optimization [17].

In order to ensure automated production of lightweight adhesive bonds with continuous in-service monitoring, a sensor position must be found that exhibits a sufficiently high sensor effect to compensate for the scattering. While recent nanocomposite-based sensing materials offer the potential to tailor sensing behavior and achieve higher gauge factors, this study employs an established silver ink to isolate and investigate the influence of sensor placement and the associated process-related challenges. This study contributes by introducing a novel external sensor placement across the adhesive gap so that maximum elongation of the sensor is achieved. These sensors are printed using the jet dispensing process and experimentally tested under quasi-static lap-shear loading and compared to sensors in a more conventional position located in the adhesive gap.

## 2. Materials and Methods

**Figure 1:**
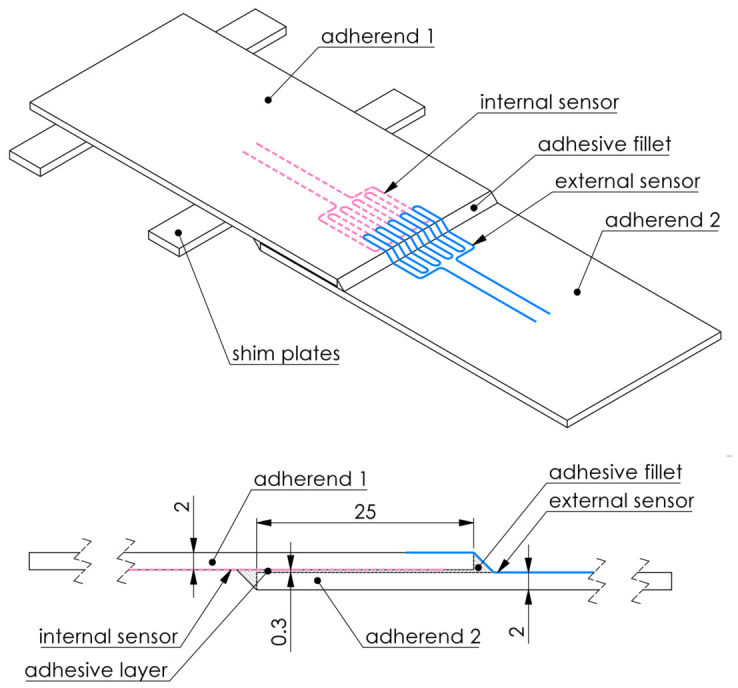
Specimen geometry design scaled up from DIN EN 1465, consisting of adherends 1 and 2 with one internal and one external printed sensor.

Tensile lap-shear tests based on DIN EN 1465 [18] were performed to investigate the new sensor position. Due to the achievable sensor size, the specimen geometry was scaled up as shown in Figure 1 to create an adhesive area of 50 × 25 mm. CFRP (carbon fiber reinforced plastic) with a thickness of 2 mm was used for adherends 1 and 2. The material composition of the CFRP is provided in Appendix A.1. The bonding surfaces were textured using DIATEX 1500EV6 tear-off fabric (Diatex, Auvergne-Rhône-Alpes, France) to increase adhesion.

The adhesive joint was created using a 0.3 mm thick adhesive layer with the two-component epoxy resin Araldite 2011 (Huntsman Advanced Materials GmbH, Basel, Switzerland). Adherend 1 was positioned with shim plates above adherend 2 as shown in Figure 1 to ensure a defined and uniform adhesive layer thickness. In addition to the adhesive bond, filets were applied manually with a dispensing gun to the ends of the adherends to enable the external sensor to transition from the upper to the lower adherend. Two sensors were integrated into each specimen. An internal sensor was printed onto adherend 1 and subsequently sintered in an oven (Nabertherm N15_65A, Nabertherm GmbH, Lilienthal, Germany) for 20 min at 125 °C in accordance with the recommendation of the silver ink manufacturer. Adherend 2 was then bonded, embedding the internal sensor within the adhesive layer. After the adhesive was cured for 48 h at 21 °C, the external sensor was printed across the adhesive gap. The entire specimen was placed in the oven again for 20 min at 125 °C to sinter the second sensor.

Additional test specimens were manufactured, some with an internal sensor rotated by 90° so that it was transverse to the direction of loading, as well as samples without a sensor. These test series are intended to examine the influence of the sensors on the lap-shear strength of the bonded specimen as well as the transverse sensitivity. The alignment of these sensors is shown in Appendix B. In total, 5 samples from each series (internal and external sensor; internal transverse sensor; reference) were tested.

**Figure 2:**
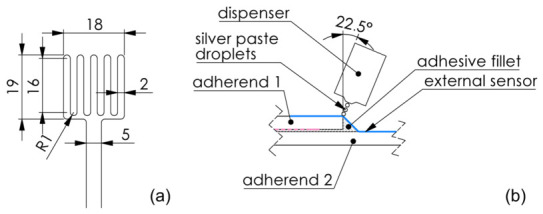
(**a**) Sensor geometry of the printed strain gauges. (**b**) Printing direction for the printing process on the sloping surface for the external sensor.

**Figure 3:**
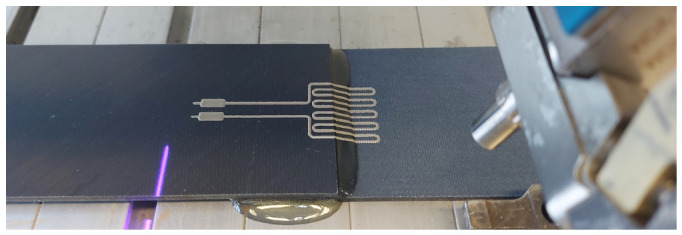
Printed external sensor with tilted jet dispenser.

To print the sensor geometry shown in Figure 2a, the jet dispensing method was used with the MDV 3250 + FC dispenser (VERMES Microdispensing GmbH, Holzkirchen, Germany) with the 1901-SB silver conductive paste (Ferro Corporation, Mayfield Heights, OH, USA) on a BZT PFE 510 PX motion system (BZT Maschinenbau GmbH, Leopoldshöhe, Germany). A complete configuration of the dispenser and the dispensing parameters can be found in Appendix A.2. For the internal sensor, the ink was dispensed vertically onto the surface, while for the external sensor, the dispenser was tilted so that a constant application angle of 22.5° was maintained on both the horizontal and sloped segments, as can be seen in the schematic in Figure 2b and the finished sensor in Figure 3. To facilitate reproducibility, the NC (Numerical Control) codes for sensor printing are listed in Appendix A.3, and the achieved base resistance of the sensors after sintering can be found in Appendix A.4.

The sensor responses are evaluated with respect to the tensile lap-shear strength τ_v_, which is calculated in accordance with DIN EN 1465 using Equation (1), given below [18]. After curing of the adhesive, the overlap length and width of the bonded specimen are measured using a caliper. Based on these values, the bonded area A is calculated.

(1) $$\tau_{v}=\frac{F_{max}}{A}\;\left[N/{\mathrm{mm}}^{2}\right]$$

**Figure 4:**
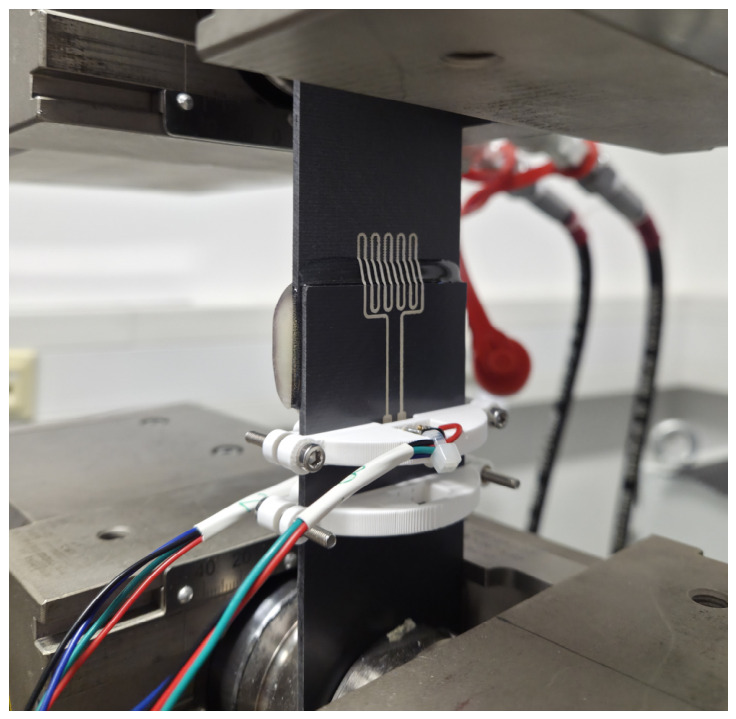
Test setup showing the specimen and externally printed sensor, the four-wire contact arrangement using spring contact pins, and the corresponding fixtures.

The tensile force $F_{max}$ was determined using an Inspekt Desk 50 tensile testing machine (Hegewald & Peschke Meß- und Prüftechnik GmbH, Nossen, Germany) with the 50 kN load cell and a crosshead speed of 1 mm/min. During the tensile test, the sensors were connected to the measuring device via spring contact pins (7911-0-15-20-86-14-11-0, Mill-Max Mfg. Corp., Oyster Bay, NY, USA [19]) in a four-wire arrangement as shown in Figure 4. Sensor resistance R was recorded using a 90-3K.3 digital ohmmeter (ELABO GmbH, Crailsheim, Germany). The measured values were transmitted from the analog voltage output of this ohmmeter to the measurement input of the tensile testing machine and measured simultaneously with the force values at a sampling rate of 50 Hz without any filtering. At the start of each test, the base resistance $R_{0}$ was measured using the same setup, the values of which can be found in Appendix A.4. The relative resistance change was calculated based on these measurements using Equation (2). In addition to the arithmetic mean and standard deviation, the coefficient of variation according to Equation (3) is used to evaluate the relative resistance change.

(2) $$relative\;resistance\;change=\frac{∆R}{R_{0}}=\frac{R-R_{0}}{R_{0}}\times 100\;[\%]$$

(3) $$coefficient\;of\;variation=\frac{standard\;deviation}{arithmetic\;mean}\times 100\;[\%]$$

## 3. Results

**Figure 5:**
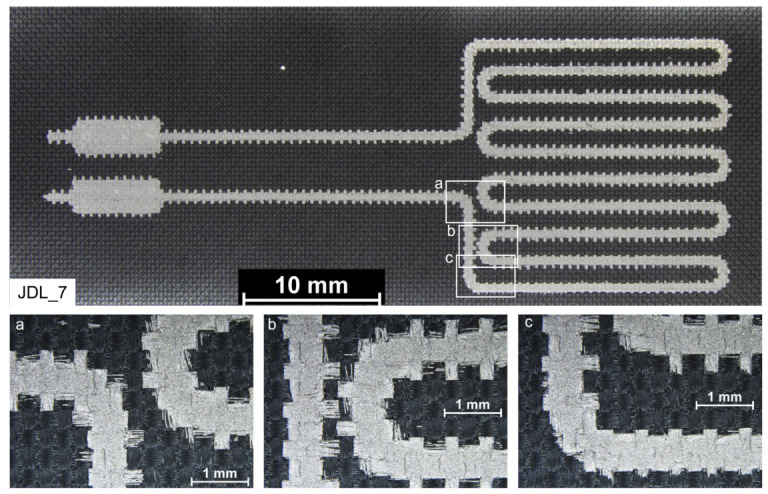
Microscopic images of the test specimens with the internal sensor printed on adherend 1. (**a**–**c**) magnified regions showing raster pattern and nearly contacting conductive tracks; positions of the zoomed areas are indicated in the overview image.

**Figure 6:**
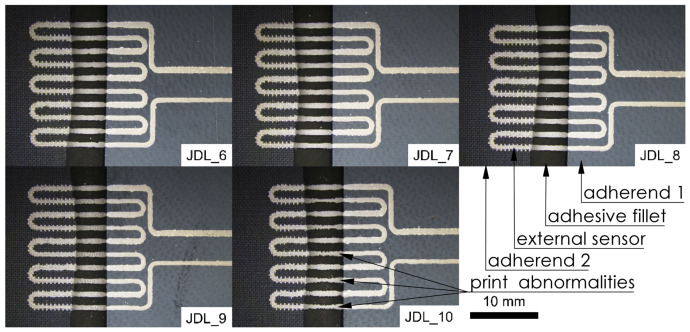
Microscopic images of the specimens with the external sensor across the adhesive gap, illustrating the variability in fillet geometry as well as abnormalities in the jet dispensing process on JDL_10.

Microscopic images of the tested specimens were acquired to check the adhesive fillet, print quality, and sensor geometry. The images in Figure 5 show the effect of the surface topography remaining from the tear-off fabric, causing the silver conductive paste to flow into this structure and leading to an inconstant conductive trace. In Figure 6, this effect is only visible on the lower inner sides of the adhesive joint. However, Figure 6 reveals that the adhesive fillet is less sloped and more inconsistent than intended in Figure 1. These inconsistencies could potentially lead to a local amplification of the sensor. Sample JDL_10 also shows abnormalities in the print path. Depending on the printing direction (slope downward or upward), the print pattern varies noticeably, as shown in Figure 6. No definite cause for this could be identified, but an inhomogeneity in the paste during this printing process is suspected.

**Figure 7:**
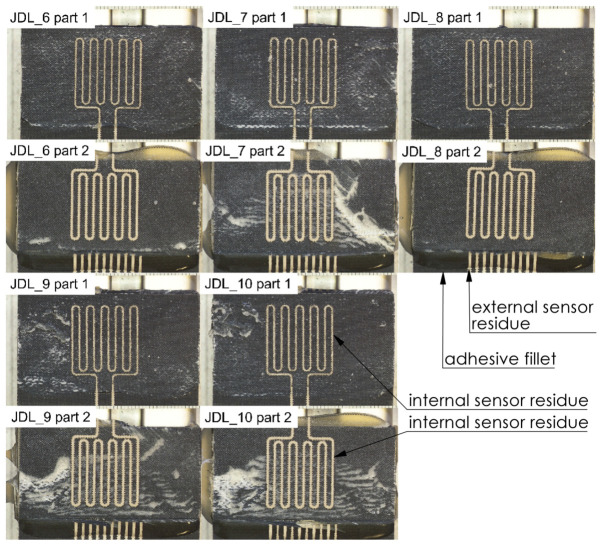
Fracture surfaces of adherends 1 and 2 of the test specimens, showing consistent adhesive failure combined with partial delamination, as well as fillet fracture edges and residual sensor material.

**Figure 8:**
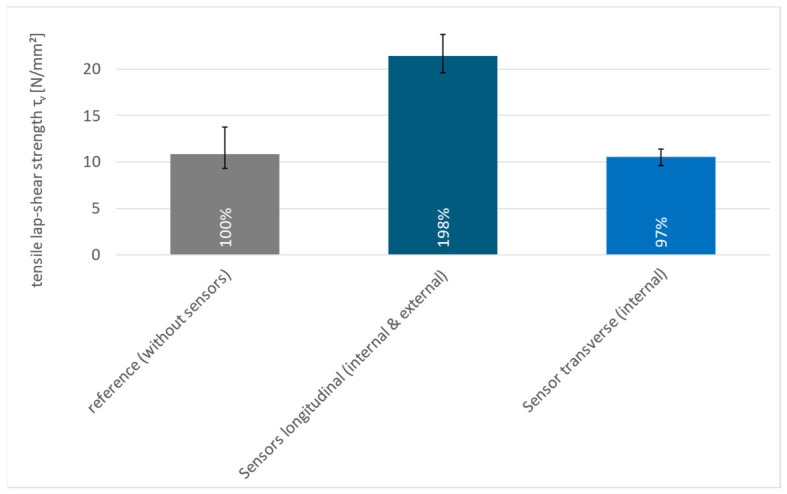
Comparison of tensile lap-shear strength for specimens without integrated sensor, with integrated transverse-aligned sensor, and with internal and external sensors. For specimens containing internal and external sensors, a second sintering step was performed with the adhesive joint already assembled, resulting in post-curing of the adhesive.

The fracture pattern shown in Figure 7 indicates consistent adhesive failure combined with partial delamination of the top adherend layer across all tested specimens. This failure consistently occurs on adherend 1, i.e., the side of the printed internal sensor. This can be seen from the sensor residues on both fracture surfaces. This indicates a weakening of the adhesive bond due to the internal sensor. However, a comparison of the tensile shear strengths achieved by the specimens with one transversely arranged internal sensor with those of reference specimens in Figure 8 shows that this weakening effect is comparatively small. The specimen containing only an internally and transversely aligned sensor reaches 97% (10.55 N/mm^2^) of the reference strength achieved by specimens without sensors (10.84 N/mm^2^). The second sintering step applied to the already-bonded specimens to cure the external sensor in specimens with internal and external sensors results in post-curing of the adhesive, leading to an increase in tensile shear strength to 198% (21.43 N/mm^2^); these specimens thus cannot be compared directly to the reference. The test series without a sensor and with a transversely printed sensor can be found in Appendix B. The external sensor breaks off at the transition from the adhesive fillet to adherend 1. The fillet with part of the sensor remains on adherend 2, as shown in Figure 7.

**Figure 9:**
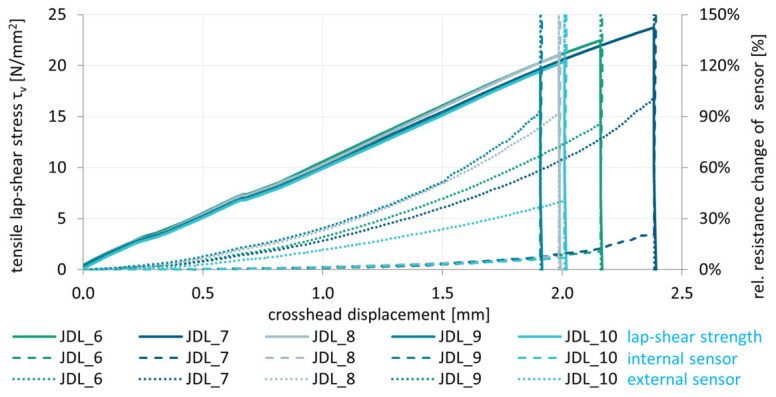
Tensile lap-shear stress–displacement plot of the lap-shear test, including the corresponding relative resistance changes in the internal and external sensors.

**Figure 10:**
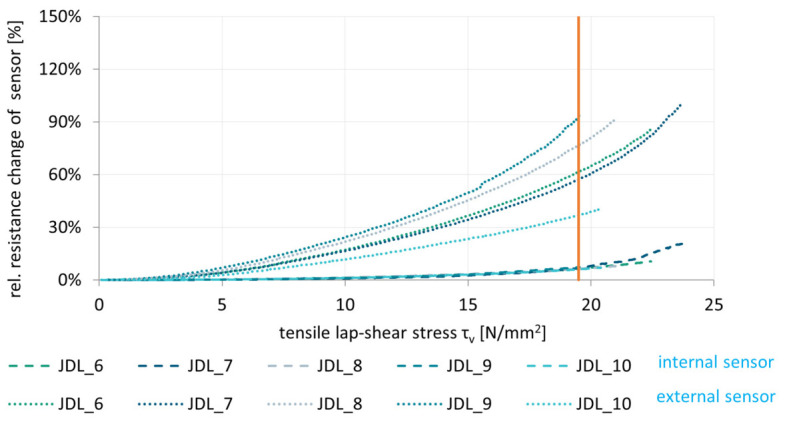
Sensor response vs. tensile lap-shear stress plot of the lap-shear test. A vertical reference line at 19.5 N/mm^2^ indicates the stress level at which the relative resistance changes from all curves were taken and summarized in Table 1.

Figure 9 shows the tensile shear stress calculated using Equation (1) together with the resulting sensor responses of the internal and external sensors. While the force and thus the calculated shear stress increase linearly at a constant test speed, the sensor response increases nonlinearly. Furthermore, the external sensors show a much higher sensor response than the internal sensors but also scatter more. For quantification, the sensor responses shown in Figure 9 were related to the calculated shear stress in Figure 10. The observed nonlinear sensor response is consistent with the external sensor position spanning the adhesive gap, where the local strain state is expected to evolve nonlinearly with increasing load due to joint deformation and progressive load transfer. As a consequence, a single gauge factor is not representative of this sensor configuration, and the sensor performance is therefore evaluated using relative resistance changes at defined shear stress levels. The lowest failure stress observed among all specimens was 19.53 N/mm^2^. Thus, a stress level of 19.5 N/mm^2^ was selected for comparison of the relative resistance change in all specimens. The corresponding values were extracted from the dataset at the first occurrence of this stress level and summarized in Table 1. As expected, the external sensor shows an average sensor response of 65.3%, which is almost 10 times higher than that of the internal sensor with 6.6% for the same stress level. At the same time, the coefficient of variation calculated according to Equation (3) is considerably higher at 32.6% for the external sensor compared to 7.6% for the internal sensor. Since the microscopic images in Figure 6 showed that sample JDL_10 exhibited abnormalities in the print quality, the same calculations were repeated excluding JDL_10. As a result, the sensor response of the external sensor increases further to an average relative resistance change of 72.4% at a shear stress of 19.5 N/mm^2^ in the adhesive joint, and the coefficient of variation is reduced to a still elevated but significantly improved value of 22.7%.

## 4. Discussion

This study successfully demonstrated the fabrication of printed strain sensors positioned across the adhesive gap, corresponding to the region of maximum expected strain. The performed tensile lap-shear tests revealed that the external sensor position yields a sensor response approximately one order of magnitude higher than that of the internal sensor position. With respect to the objective of achieving a sensor response sufficiently large to compensate for resistance changes caused by environmental influences, the external sensor position proves advantageous. The observed average relative resistance change of 65.3% near the failure load (Table 1) clearly exceeds the environmentally induced resistance variations of up to 19% reported in the literature for printed strain sensors. The proposed sensor configuration therefore is a promising candidate for condition-monitoring applications. However, it should be noted that the present study is limited to quasi-static loading conditions and does not yet demonstrate reliable damage detection under cyclic loading conditions.

Recent approaches for adhesive joint monitoring include self-sensing systems, where adhesives are mixed with conductive additives, which allows monitoring of joints through changes in electrical properties of the adhesive layer itself and the interfacial contact with the adherends due to crack propagation [20,21]. These approaches allow a rather global assessment of structural integrity. In contrast, back-face strain measurements capture highly localized changes in the strain field and can resolve crack initiation and propagation with high spatial precision through discrete sensor placement [4,5,6,7]. The approach presented in this work lies between these concepts. By placing a printed sensor across the adhesive gap in a region of presumed maximum strain, changes in the local deformation state can be detected at a defined position along a larger joint. While the spatial resolution is lower than for distributed back-face strain sensors, the approach provides more localized information than global self-sensing methods without requiring modification of the adhesive material.

Future work could combine the identified sensor position with advanced sensing materials, such as nanocomposite-based systems discussed in the Introduction, to further increase the achievable sensitivity. In addition, reducing the observed measurement scatter remains essential. In particular, a well-defined and uniformly reproducible adhesive fillet is required to ensure a consistent sensor geometry and therefore improved measurement repeatability. To achieve this, future work should employ an automated, position- and volume-accurate dispensing system instead of manual application for the adhesive joint.

## Figures and Tables

**Table 1:** Relative resistance changes in the internal and external sensors at 19.5 N/mm^2^ for all specimens, with calculated mean, standard deviation, and coefficient of variation.

	Rel. Resistance Change at 19.5 N/mm^2^ [%]					Arithmetic Mean [%]	Standard Deviation [%]	Coefficient of Variation [%]
	JDL_6	JDL_7	JDL_8	JDL_9	JDL_10			
Internal sensor	6.3	6.9	6.3	7.3	6.1	6.6	0.50	7.6
External sensor	61.7	57.3	77.0	93.5	36.9	65.3	21.3	32.6

**Figure A1:**
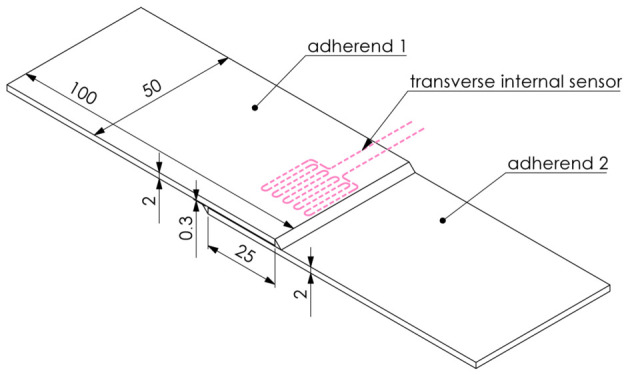
Sample geometry design scaled up from DIN EN 1465, consisting of adherends 1 and 2 with a transversely oriented internal printed sensor.

**Figure A2:**
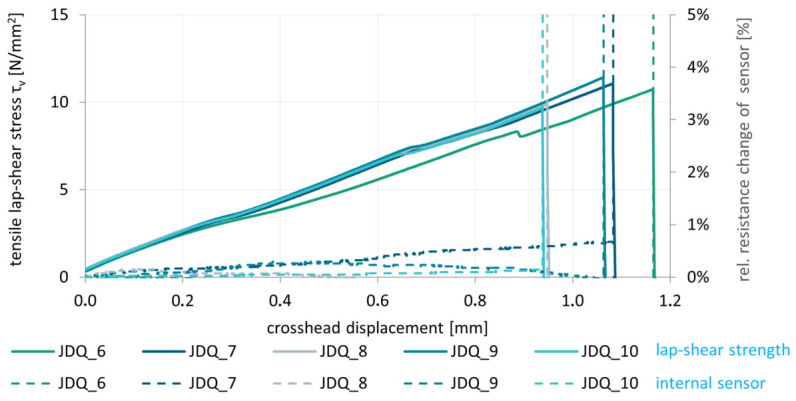
Lap-shear stress–displacement plot of the lap-shear test with relative resistance changes in the transversely oriented internal sensors.

**Figure A3:**
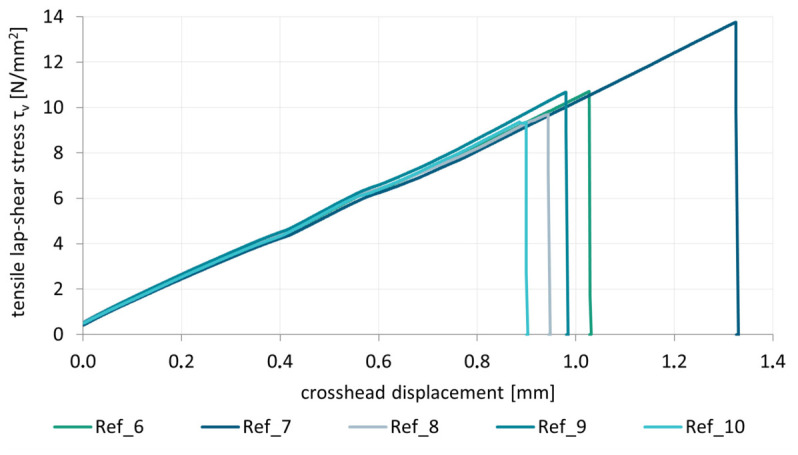
Lap-shear stress–displacement plot of the lap-shear test of the reference specimens without sensors.

**Figure A4:**
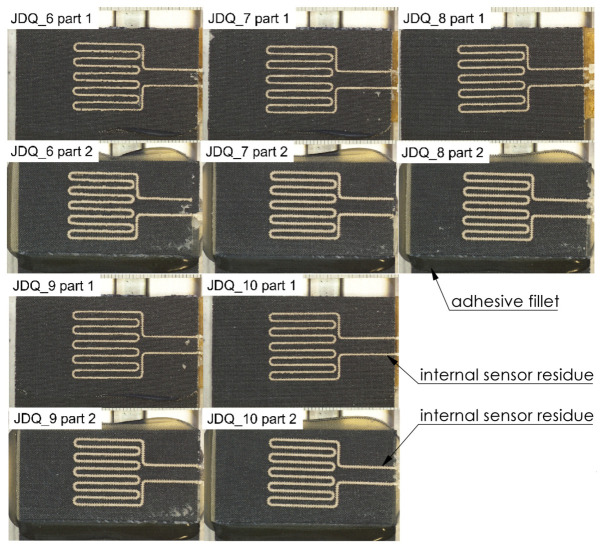
Fracture surfaces of the test specimens with transversely oriented internal printed sensor.

## Data Availability

The original contributions presented in this study are included in the article. Further inquiries can be directed to the corresponding author.

## Abbreviations

The following abbreviations are used in this manuscript:

CFRP	Carbon-fiber-reinforced plastics
GF	Gauge factor
NC	Numerical control

## Appendix A

### Appendix A.1

The CFRP samples used were manufactured using a prepreg and a DIATEX 1500EV6 peel ply on the adhesive surfaces. The outermost layer of the laminate was made with glass fiber to create a non-conductive surface. The material properties are shown in Table A1.

**Table A1:** Material properties of the CFRP.

Prepreg	
Weight per unit area	210 g/m^2^
Carbon fiber	
Fiber diameter	7 µm
Fiber density	1.8 g/cm^3^
Orientation	unidirectional
Matrix	
Type	Epoxy resin
Matrix density	1.23 g/cm^3^
Glass transition temperature	125 °C

### Appendix A.2

The sensors were printed using the dispenser MDV 3250 + FC from VERMES Microdispensing with the silver paste 1901-SB from Ferro GmbH. The complete configuration of the dispenser is listed in Table A2.

**Table A2:** Dispenser configuration.

Hardware	
Dispenser	MDV 3250 + FC
Piston rod	TTF 15 + 10 mm
Nozzle diameter	100 µm (N11-100)
Piston seal	LX
Parameters	
Cartridge pressure	2.5 bar
Dispenser cooling pressure	2 bar
Dispenser heating	40 °C
Rising time	0.21 ms
Opening time	1.4 ms
Falling time	0.07 ms
Needle lift	70%
Number of pulses	1
Delay	0.1 ms
Jet shot density	4
Distance from specimen	2 mm

### Appendix A.3

**Table A3:** NC-Code for sensor printing.

Internal Sensor	External Sensor
G90 (Absolute coordinates) G94 (Feed rate in mm/min) G17 (X-Y plane) G21 (Metric system) G54 (Work coordinate system 1) G0 Z20 F1200 G0 X27.278014 Y53.459015 Z20 G0 Z0 M54P5 (Dispenser-ON) G1 F1200 G1 X27.278014 Y23.459015 G3 X28.278014 Y22.459015 I1 J0 G1 X33.028014 Y22.459015 G2 X34.028014 Y21.459015 I0 J-1 G1 X34.028014 Y20.459015 G1 X34.028014 Y4.459015 G2 X32.028014 Y4.459015 I-1 J0 G1 X32.028014 Y20.459015 G3 X30.028014 Y20.459015 I-1 J0 G1 X30.028014 Y4.459015 G2 X28.028014 Y4.459015 I-1 J0 G1 X28.028014 Y20.459015 G3 X26.028014 Y20.459015 I-1 J0 G1 X26.028014 Y4.459015 G2 X24.028014 Y4.459015 I-1 J0 G1 X24.028014 Y20.459015 G3 X22.028014 Y20.459015 I-1 J0 G1 X22.028014 Y4.459015 G2 X20.028014 Y4.459015 I-1 J0 G1 X20.028014 Y20.459015 G3 X18.028014 Y20.459015 I-1 J0 G1 X18.028014 Y4.459015 G2 X16.028014 Y4.459015 I-1 J0 G1 X16.028014 Y20.459015 G1 X16.028014 Y21.459015 G2 X17.028014 Y22.459015 I1 J0 G1 X21.778014 Y22.459015 G3 X22.778014 Y23.459015 I0 J1 G1 X22.778014 Y53.459015 M55P5 (Dispenser-OFF) (Pads) G91 G1 Y-2 X-1 M54P5 (Dispenser-ON) G1 Y-6 G1 X0.5 G1 Y6 G1 X1 G1 Y-6 G1 X0.5 G1 Y6 M55P5 (Dispenser-OFF) G90 G0 X27.278014 Y53.459015 G91 G1 Y-2 X-1 M54P5 (Dispenser-ON) G1 Y-6 G1 X0.5 G1 Y6 G1 X1 G1 Y-6 G1 X0.5 G1 Y6 M55P5 (Dispenser-OFF) G90 M55P5 (Dispenser-OFF) G0 Z20 G0 X0 Y0 M30	G90 (Absolute coordinates) G94 (Feed rate in mm/min) G17 (X-Y plane) G21 (Metric system) G54 (Work coordinate system 1) G0 Z20 F1200 G0 X65.959015 Y22.721986 G0 Z0 M54P5 (Dispenser-ON) G1 F1200 G1 X35.959015 Y22.721986 G3 X34.959015 Y21.721986 I0 J-1 G1 X34.959015 Y16.971986 G2 X33.959015 Y15.971986 I-1 J0 G1 X32.959015 Y15.971986 G1 X29 Y15.971986 G1 X23 Y15.971986 Z-2 G1 X17.789015 Y15.971986 G2 X17.789015 Y17.971986 I0 J1 G1 X23 Y17.971986 G1 X29 Y17.971986 Z0 G1 X32.959015 Y17.971986 G3 X32.959015 Y19.971986 I0 J1 G1 X29 Y19.971986 G1 X23 Y19.971986 Z-2 G1 X17.789015 Y19.971986 G2 X17.789015 Y21.971986 I0 J1 G1 X23 Y21.971986 G1 X29 Y21.971986 Z0 G1 X32.959015 Y21.971986 G3 X32.959015 Y23.971986 I0 J1 G1 X29 Y23.971986 G1 X23 Y23.971986 Z-2 G1 X17.789015 Y23.971986 G2 X17.789015 Y25.971986 I0 J1 G1 X23 Y25.971986 G1 X29 Y25.971986 Z0 G1 X32.959015 Y25.971986 G3 X32.959015 Y27.971986 I0 J1 G1 X29 Y27.971986 G1 X23 Y27.971986 Z-2 G1 X17.789015 Y27.971986 G2 X17.789015 Y29.971986 I0 J1 G1 X23 Y29.971986 G1 X29 Y29.971986 Z0 G1 X32.959015 Y29.971986 G3 X32.959015 Y31.971986 I0 J1 G1 X29 Y31.971986 G1 X23 Y31.971986 Z-2 G1 X17.789015 Y31.971986 G2 X17.789015 Y33.971986 I0 J1 G1 X23 Y33.971986 G1 X29 Y33.971986 Z0 G1 X32.959015 Y33.971986 G1 X33.959015 Y33.971986 G2 X34.959015 Y32.971986 I0 J-1 G1 X34.959015 Y28.221986 G3 X35.959015 Y27.221986 I1 J0 G1 X65.959015 Y27.221986 M55P5 (Dispenser-OFF) (Pads) G91 G1 Y-1 X-2 M54P5 (Dispenser-ON) G1 X-6 G1 Y0.5 G1 X6 G1 Y1 G1 X-6 G1 Y0.5 G1 X6 M55P5 (Dispenser-OFF) G90 G0 X65.959015 Y22.721986 G91 G1 X-2 Y-1 M54P5 (Dispenser-ON) G1 X-6 G1 Y0.5 G1 X6 G1 Y1 G1 X-6 G1 Y0.5 G1 X6 M55P5 (Dispenser-OFF) G90 M55P5 (Dispenser-OFF) G0 Z20 G0 X0 Y0 M30

### Appendix A.4

After sintering the sensors according to the process described in the Section 2 and connecting them using the spring contact pins, the sensors exhibited the base resistances shown in Table A4. These were measured just before the tensile test using the measuring device described in the Section 2.

**Table A4:** Base resistance of the printed sensors.

	**Base Resistance [Ω]**				
	**JDL_6**	**JDL_7**	**JDL_8**	**JDL_9**	**JDL_10**
Internal sensor	8.13	7.06	8.03	7.19	7.12
External sensor	12.11	11.66	12.35	12.51	7.65
	**JDQ_6**	**JDQ_7**	**JDQ_8**	**JDQ_9**	**JDQ_10**
Transverse internal sensor	7.08	7.41	8.01	6.83	8.22

## Appendix B

Additional test specimens with internal sensors transverse to the direction of loading (results shown in Figure A2) and without sensors (results shown in Figure A3) were manufactured. The dimensions of the adherends and the design of the adhesive bond are the same as those explained in Figure 1. The transversely aligned sensor is shown in Figure A1. The fracture pattern of the transversely printed sensors in Figure A4 shows adhesive failure on the side of the printed sensor, similar to the longitudinally printed specimens, as evidenced by the sensor residues on both adhesive partners.
